# Genomic surveillance of severe acute respiratory syndrome coronavirus 2 in Burundi, from May 2021 to January 2022

**DOI:** 10.1186/s12864-023-09420-3

**Published:** 2023-06-10

**Authors:** Cassien Nduwimana, Néhémie Nzoyikorera, Armstrong Ndihokubwayo, Théogène Ihorimbere, Célestin Nibogora, Adolphe Ndoreraho, Oscar Hajayandi, Jean Claude Bizimana, Idrissa Diawara, Dionis Niyonizigiye, Joseph Nyandwi

**Affiliations:** 1 National Reference Laboratory, National Institute of Public Health, Bujumbura, Burundi; 2 Mohamed VI University of Health Sciences (UM6SS), Higher Institute of Biosciences and Biotechnology, Casablanca, Morocco; 3 Mohamed VI Center for Research & Innovation, Laboratory of Microbial Biotechnology and Infectiology Research, Mohamed VI University of Health Sciences (UM6SS), Rabat, Morocco; 4 National Institute of Public Health, Ministry of Public Health and the Fight against AIDS, Bujumbura, Burundi; 5 Public Health Emergency Operation Center, Ministry of Public Health and the Fight against AIDS, Bujumbura, Burundi; 6 Faculté de Médecine, Université du Burundi, Bujumbura, Burundi

**Keywords:** COVID-19, SARS-COV-2 variants, Waves, Genetic diversity, Lineage, Amino acid change

## Abstract

**Background:**

The emergence and rapid spread of new severe acute respiratory syndrome coronavirus 2 (SARS-COV-2) variants have challenged the control of the COVID-19 pandemic globally. Burundi was not spared by that pandemic, but the genetic diversity, evolution, and epidemiology of those variants in the country remained poorly understood. The present study sought to investigate the role of different SARS-COV-2 variants in the successive COVID-19 waves experienced in Burundi and the impact of their evolution on the course of that pandemic.

We conducted a cross-sectional descriptive study using positive SARS-COV-2 samples for genomic sequencing. Subsequently, we performed statistical and bioinformatics analyses of the genome sequences in light of available metadata.

**Results:**

In total, we documented 27 PANGO lineages of which BA.1, B.1.617.2, AY.46, AY.122, and BA.1.1, all VOCs, accounted for 83.15% of all the genomes isolated in Burundi from May 2021 to January 2022. Delta (B.1.617.2) and its descendants predominated the peak observed in July–October 2021. It replaced the previously predominant B.1.351 lineage. It was itself subsequently replaced by Omicron (B.1.1.529, BA.1, and BA.1.1). Furthermore, we identified amino acid mutations including E484K, D614G, and L452R known to increase infectivity and immune escape in the spike proteins of Delta and Omicron variants isolated in Burundi. The SARS-COV-2 genomes from imported and community-detected cases were genetically closely related.

**Conclusion:**

The global emergence of SARS-COV-2 VOCs and their subsequent introductions in Burundi was accompanied by new peaks (waves) of COVID-19. The relaxation of travel restrictions and the mutations occurring in the virus genome played an important role in the introduction and the spread of new SARS-COV-2 variants in the country. It is of utmost importance to strengthen the genomic surveillance of SARS-COV-2, enhance the protection by increasing the SARS-COV-2 vaccine coverage, and adjust the public health and social measures ahead of the emergence or introduction of new SARS-COV-2 VOCs in the country.

**Supplementary Information:**

The online version contains supplementary material available at 10.1186/s12864-023-09420-3.

## Background

The COVID-19 pandemic that started in Wuhan, China in December 2019 [1] rapidly spread globally causing millions of cases and taking the lives of millions of people [2]. As of 4 January 2023, over 656 million cases and over 6 million deaths had been reported globally [3, 4]. In Africa, the first case was reported on February 14, 2020 in Egypt, and by January 2023 the African continent had recorded over 9 million COVID-19 cases and 175,140 deaths. This pandemic did not spare Burundi. The first two cases of COVID-19 were detected in Burundi on March 31, 2020, prompting the public health authorities to put in place control measures. These cases were national citizens returning from countries that had experienced COVID-19 before Burundi, including the United Arab Emirates and the Republic of Rwanda. As of January 11, 2021, the National Committee on COVID-19 fighting implemented a ban on international passenger flights and terrestrial travel. As of April 1, 2021, this restriction was lifted and replaced by a 7-day quarantine for all passengers entering the country [5]. Further control measures included mask-wearing, keeping physical distancing in public places, hand hygiene, free COVID-19 screening, and the COVID-19 vaccination [6, 7]. Burundi experienced a resurgence of COVID-19 in July 2021, with an increase in the number of new cases reaching 37.26% of previously reported cases. Most of these new cases were reported in Kiremba and Kirundo health districts as well as the central district of the municipality of Bujumbura [7]. Despite the substantial drop in COVID-19 cases observed in November 2021 – following the public health and social measures put in place – a sharp re-increase in COVID-19 cases was observed from December 2021. This wave was marked by the highest peak of cases ever recorded in Burundi since the first report of COVID-19 cases in the country [8]. By December 2022, a total of 52,162 COVID-19 confirmed cases and 15 associated deaths had been reported in Burundi since the first report of two imported COVID-19 cases [9].

Globally, the emergence of new SARS-COV-2 variants challenged the public health interventions aimed at controlling the pandemic [10–12]. Different SARS-COV-2 variants of concern (VOC) including Alpha variant (B.1.1.7), Beta variant (B.1.351), Gamma variant (P.1), Delta variant (B.1.617.2), and Omicron variant (B.1.1.529) and their sublineages spread globally [11, 13], entrenching the difficulties to control this pandemic.

It is of utmost importance to continuously monitor locally circulating SARS-COV-2 lineages to allow timely detection of new variants before widespread local and international dissemination [14]. In Burundi, the genetic diversity and the evolution of SARS-COV-2 variants that puzzled the control of the COVID-19 pandemic remained undocumented. The present study sought to document the genetic diversity of SARS-COV-2 lineages circulating in Burundi, their evolution, and their role in different COVID-9 waves experienced in that country.

## Results

### Demographic characteristics of the study participants

The age of the study participants ranged between 1 and 94 years (mean age = 32.19, standard deviation = 17.64) and 56.14% (210/374) were male while females represented 43.86% (164/374). They were distributed across different provinces of the country including the municipality of Bujumbura (*n* = 198), the Province of Bujumbura – formerly known as rural Bujumbura – (*n* = 39), Kirundo (*n* = 55), Ngozi (*n* = 35), Muyinga (*n* = 11), Gitega (*n* = 10), Rutana (*n* = 5), Bururi (*n* = 4), Kayanza (*n* = 4), Makamba (*n* = 3), Mwaro (*n* = 3), Bubanza (*n* = 2), Ruyigi (*n* = 2), and Rumonge (*n* = 1).

### The genetic diversity of the SARS-COV-2 lineages detected in Burundi from May 2021 to January 2022

Overall, 374 SARS-COV-2 genomes were successfully sequenced in the reference sequencing laboratory of Uganda (Uganda Virus Research Institute) and Senegal (Institut Pasteur de Dakar), from May 2021 to January 2022. Of the 374 genomes, 239 (63.9%) were sequenced using Illumina and 135 (36.1%) using the MinIon sequencing platform (Additional File 1).

**Fig. 1:**
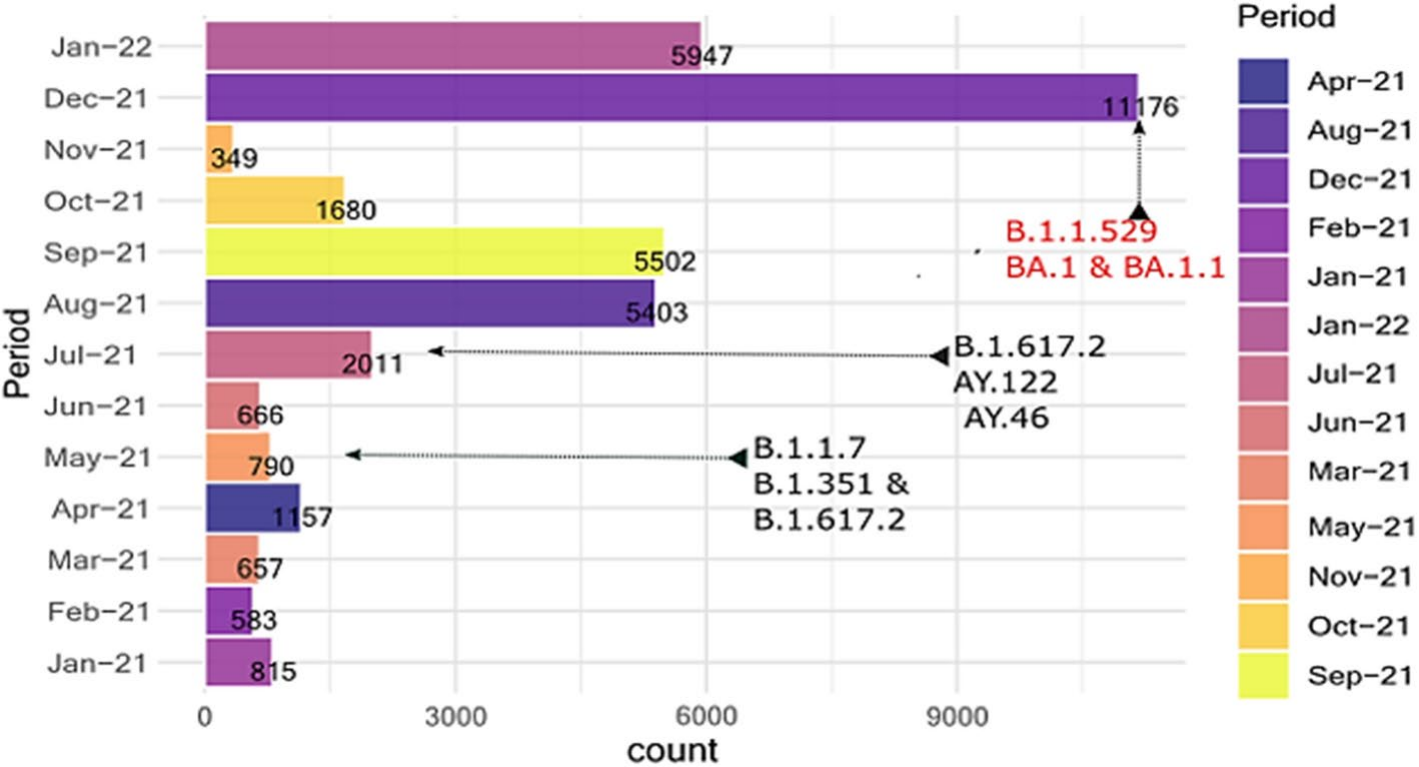
Distribution of COVID-19 confirmed cases in 2021 and the SARS-COV-2 lineage composition of different COVID-19 waves. This bar plot shows the number of laboratory-confirmed COVID-19 cases documented in Burundi during the present study period and the SARS-COV-2 lineages involved in specific COVID-19 waves. B.1.1.7 = Alpha variant, B.1.351 = Beta variant, B.1.617.2 = Delta variant, B.1.1.529 = Omicron variant, BA.1 and BA.1.1 are Omicron sublineages, AY. 122 & AY.46 are Delta sublineages

**Fig. 2:**
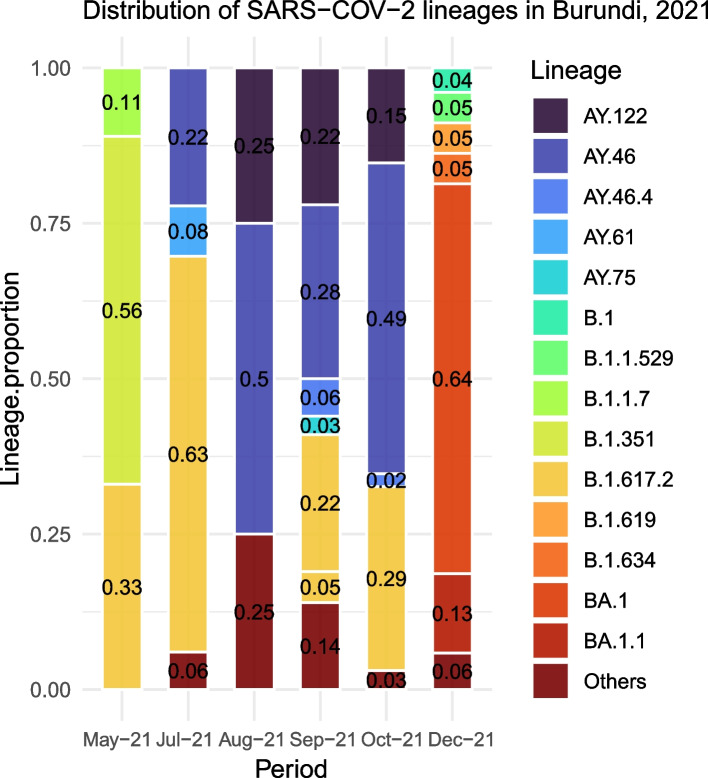
Distribution of different SARS-COV-2 lineages involved in COVID-19 infections in Burundi from May to December 2021. This graph captures the monthly proportions of different SARS-COV-2 lineages identified in Burundi from May to December 2021

Two different peaks of COVID-19 cases – from July to November 2021 and December 2021 to January 2022 respectively – predominated by different SARS-COV-2 lineages were observed during the present study period (Fig. 1). The SARS-COV-2 lineage B.1.617.2 (Delta) was predominant during the wave that occurred from July to October 2021 and accounted for 63.2% (*n* = 55) of all the SARS-COV-2 genomes isolated in Burundi during that period. On the other hand, the peak of cases observed from December 2021 was predominated by the lineage B.1.1.529 (Omicron) and its sublineages BA.1 and BA.1.1, accounting for ~ 82% of all the SARS-COV-2 genomes identified in Burundi during that period (Fig. 2). Clearly, the Omicron variant tended to replace the Delta (B.1.617.2) lineage which represented only 1% of all the SARS-COV-2 lineages identified in December 2021 and January 2022.

The Omicron lineage (B.1.1.529) and its sublineages BA.1 and BA.1.1 were first identified in Burundi from samples collected on November 24, 2021 (not shown on the figure). The identification of the Omicron variant in Burundi, from samples collected from travelers and community clusters members, coincided with the occurrence of the most striking peak of COVID-19 cases ever seen in Burundi, since the introduction of that pandemic in the country (Fig. 1).

The 374 genomes sequenced in Burundi during the present study period (from January 2021 to January 2022) were classified into 27 SARS-COV-2 PANGO lineages. Of the 27 PANGO lineages, 5 accounted for 83.15% of all the SARS-COV-2 genomes sequenced in Burundi during this study period. These lineages included BA.1 (25.9%, 97/374), B.1.617.2 (Delta) lineage (24.9%, 93/374), AY.46 (20.9%, 79/374), AY.122 (6%, 23/374), and BA.1.1 (5.3%, 20/374). All 5 common PANGO lineages were variants of concern (VOCs) or their sublineages. The lineages that accounted for less than 1% per month (AY.88, AY.45, AY.47, B.1.1.294, B.1.619, C.37.1, AY.5, AY.52, AY.61, AY.70, AY.75) in our study were combined in one group referred to as “others” (Fig. 2).

### Predominant amino acid changes identified in different SARS-COV-2 lineages isolated in Burundi from May 2021 to January 2022

The most common mutations carried by different SARS-COV2 variants identified in Burundi concerned mainly the surface glycoprotein, N, and different non-structural proteins (NSP2, 3,4,5, 6, 12,13, and 14). The mutation D614G in the spike protein was shared by almost all the SARS-COV-2 genomes identified in the present study, irrespective of the lineage. Furthermore, all the Omicron (B.1.1.529, BA.1, and BA.1.1) genome sequences displayed the mutations D796Y, N764K, N856K, T547K, N696K, and N969K. Despite their presence in BA.1 and BA.1.1 genome sequences, the amino acid mutations L981F and G142D have not been identified in B.1.1.529 genomes from this study. The mutation G142D was rather shared with the Delta (B.1.617.2) variant. The following mutations have been identified in the Delta variant’s spike protein: A22V, L452R, D950N, E156G, G142D, P681R, S698L, T19R, R158del, and F157 del. Concerning the Alpha and Beta variants, their surface glycoprotein harbored the following mutations: D614G, N501Y, D215G, E484K, K417N, A701V, P681H, D1118H, S982A, L18F, T716I, L54F, and L244del (Additional file 2).

### Phylogenetic analysis

**Fig. 3:**
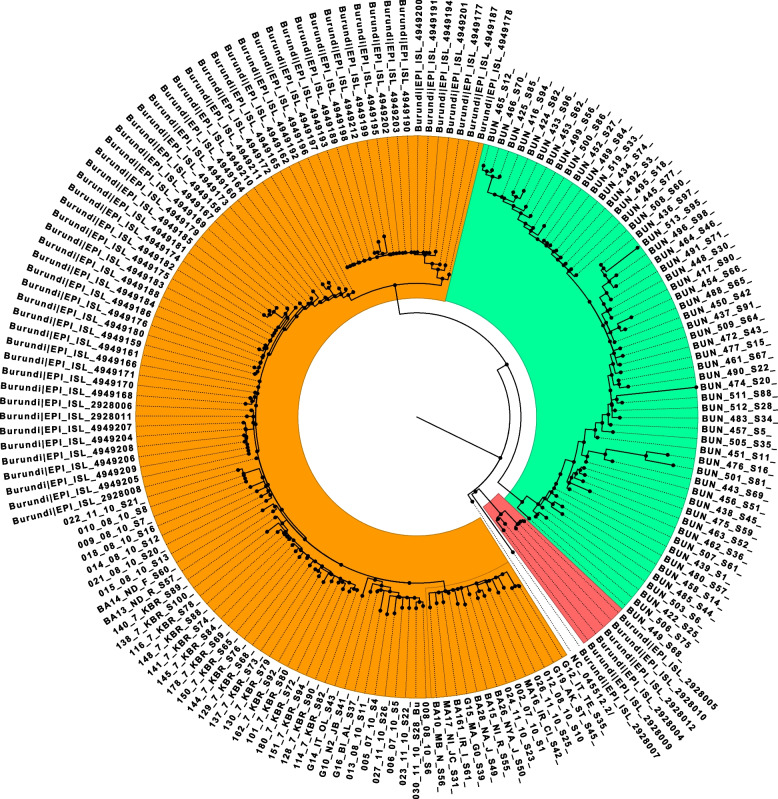
Maximum Likelihood phylogenetic tree of the nucleotide sequences of SARS-COV-2 genomes detected in Burundi in 2021. The B.1.351 genomes (*n* = 5) are indicated in pink, Delta (B.1.617.2) and its sublineages (*n* = 109) in orange, the Omicron (B.1.1.529) and its sublineages BA.1 and BA.1.1 genomes (*n* = 57) in green, and the only one B.1.1.7 genome was not colored. The reference SARS-COV-2 genome used was the SARS-CoV-2 isolate Wuhan-Hu-1, accession number NC_45512.2, retrieved from the GenBank database

The phylogenetic analysis of the sequences from SARS-COV-2 lineages identified in Burundi showed that B.1.1.7 and B.1.351 lineages clusters did not expand largely through community transmission. The B.1.1.7 lineage from this study is represented by one genome detected from a sample collected on May 27, 2021, in the municipality of Bujumbura. The SARS-COV-2 genomes detected in Burundi during that period were mostly dominated by the B.1.617.2 lineage. Despite the relatively small proportion of the B.1.351 lineage detected in this study, the 5 corresponding sequences (indicated in pink on Fig. 3) formed two phylogenetically separate clusters, suggesting two different introductions events. They were all generated from COVID-19 samples collected at different COVID-19 routine testing sites located in the municipality of Bujumbura, during the period from May 27 to May 31, 2021.

The Delta variant (highlighted in orange) was first detected in Burundi from samples collected in Kiremba health district as of May 26, 2021, during a campaign of mass COVID-19 testing. The sequences of Delta lineages from this study formed separate clusters based on different sublineages. Furthermore, the sequences of Delta genomes from Kiremba health district (sequence names in “KBR”) formed a separate cluster, suggesting a different introduction of that variant in Burundi. There have been 4 distinct phylogenetic clusters within the sublineage AY.46 highlighting substantial genetic divergence within this sublineage (Fig. 3).

However, a close genetic relatedness was observed between the sequences from the Delta genome identified in the municipality of Bujumbura and other provinces of Burundi (genomes 013_08_10_S11 & G16_BI_AL_S37 collected in the municipality of Bujumbura and Gitega Province respectively, genomes BA10_MB-N_S56 & MA17_NI-JC_S31 from the municipality of Bujumbura and Makamba Province respectively). Phylogeny reconstruction demonstrated that the COVID-19 cases observed in Kiremba health district during the June-October 2021 peak were interlinked and resulted in the expansion of initial cases, suggesting a community transmission of the pandemic within this health district.

## Discussion

The present study aimed at investigating the genetic diversity, evolution, and molecular epidemiology of SARS-COV-2 variants in Burundi. It allowed to demonstrate the great genetic diversity of SARS-COV-2 lineages that circulated in Burundi during the period from May 2021 to January 2022, with 27 lineages in total, of which 5 were VOCs. This genetic diversity was slightly lower than in other East African countries like Kenya where they detected 32 PANGO lineages. The probable sources of this inconsistency are the differences in the sample sizes used in our respective studies, the size of the target population, the intensity of international travel in the country, and the duration of the study (12 months in Kenya against 9 months in our study) [15]. Furthermore, the findings from this study demonstrated that different peaks of COVID-19 cases that followed phases of quasi-controlled COVID-19 pandemic in Burundi involved different SARS-COV-2 variants; suggesting multiple introductions of different SARS-COV-2 lineages from other countries. This is the case of the peak of COVID-19 cases observed from July to October 2021 and the peak associated with the Omicron variant from December 2021.

The introduction of B.1.617.2 (Delta) in Burundi – first detected in the country in May 2021 – and the subsequent resurgence of COVID-19 cases observed in July 2021 accompanied the lifting of movement restrictions and the reduction in quarantine duration for international travelers from 7 to 4 days in April 2021. Similar epidemiological trends after the relaxation of travel restrictions have been described elsewhere [16]. In addition to the implications of public health measures in the transmission dynamics of the COVID-19, the virus properties might have contributed to the increased transmission rates associated with Delta. This lineage carried amino acid mutations such as T19R, and G142D in the N-Terminal Domain (NTD) known to be a point of recognition for vaccines and the immune response [17]. The mutations in that region offer immune escape possibilities to the strains acquiring them. Moreover, other mutations such as E484K, D614G, and L452R known to have a substantial impact on the virus infectivity [18, 19] were identified in the Receptor-Binding Domain (RBD) of Delta genome sequences. The E484K mutation is believed to reduce the binding strength to monoclonal antibodies and to increase the binding affinity toward the angiotensin-converting enzyme 2 (ACE2) receptor [20]. These mutations have been described in previous studies [21, 22].

In addition, the present study demonstrated how the introduction of new SARS-COV-2 lineages challenged the public health interventions and social measures put in place to tackle the COVID-19 pandemic in Burundi. Similar impacts of the emergence of new variants were reported elsewhere [12]. The Omicron variant B.1.1.529 was associated with the greatest number of COVID-19 cases in Burundi and tended to replace the Delta variant which predominated in previous peaks. A similar situation was reported elsewhere and was suggested to be associated with immune escape from this variant rather than intrinsic higher transmissibility. Following its emergence, this variant of concern spread rapidly globally and became the most predominant SARS-COV-2 variant worldwide [23]. The Omicron variant genomes harbor several mutations in the spike protein which increased its ability to escape the immune response and the infectivity [19, 20, 24]. Some of these mutations were shared with Delta and included D614D, and G142D in the spike protein (S). The spike protein (S) is the major determinant of host cell tropism in coronaviruses making it the most important site for mutations and enhancing immune evasion. The D614G mutation is a common mutation that was harbored by around 84.2% of the African SARS-COV-2 isolates and reported as a predominant mutation in other African countries including Morocco, Algeria, and the Democratic Republic of Congo [15, 21, 22, 25, 26].

The phylogenetic analysis of SARS-COV-2 genomes offered an opportunity to visualize the clustering patterns of different SARS-COV-2 genomes identified in Burundi and the evolutionary relationships linking the SARS-COV-2 genomes detected in the community and those imported from other countries. In this regard, B.1.617.2 (Delta) and its sublineages AY.122 and AY.46 on one hand, and B.1.1.529 (Omicron) and its sublineage BA.1 on the other hand formed several separate clusters suggesting multiple introductions in Burundi, as a consequence of human mobility. The Omicron variant genomes from this study formed at least 4 distinct clusters. However, a close evolutionary relationship was observed between locally detected and imported genomes from travelers and national citizens returning from the Democratic Republic of Congo, Rwanda, Uganda, and Canada among others. This suggests that this variant was imported from other countries that had experienced it before Burundi. Similar genetic relatedness between SARS-COV-2 genomes from different countries was described in previous studies conducted elsewhere. These studies included one where Indian SARS-COV-2 strains showed the closest genetic relatedness with strains from the United States or China [27].

The Delta variant was first reported in India in March 2021 from December 2020 samples [25]. This lineage was first detected in Burundi from samples collected in the municipality of Bujumbura as of May 27, 2021. Further cases associated with B.1.617.2 VOC and its sublineages AY.122 and AY.46 were subsequently detected in other provinces of Burundi as a result of inter-province transmission, as suggested by the evolutionary relationships documented through the phylogeny reconstruction. It expanded through community transmission and caused a country-wide peak of COVID-19 cases observed in August- September 2021, highlighting the difficulties to control the spread of a new SARS-COV-2 variant once introduced into a susceptible population.

Nonetheless, the clustering pattern of Delta sequences detected in Kiremba health district suggests a separate introduction of that lineage. In July 2021, two separate clusters of COVID-19 cases occurred in Kiremba and Kirundo health districts located in Ngozi and Kirundo Provinces both bordering the Republic of Rwanda. Its occurrence in Burundi coincided with a global predominance and increasing spread of the variant Delta [28]. The B.1.617.2 (Delta) lineage was gradually replaced by Omicron (B.1.1. 529) starting in December 2021. Omicron caused the highest number of COVID-19 cases ever observed in Burundi since the initial phase of that pandemic. That trend was consistent with the anticipations made by public health experts about a rapid expansion of COVID-19 cases associated with the Omicron variant, based on the genomic properties of the virus [21].

As a complement to the contact tracing efforts, the phylogenetic analysis allowed us to visualize cases of community transmission of SARS-COV-2 lineages and the involvement of imported SARS-COV-2 lineages. However, this situation needs to be interpreted cautiously, especially outside the municipality of Bujumbura as insufficient sampling in those provinces might mask possible inter-province transmission events and separate introductions of SARS-COV-2 [29].

### Conclusion and recommendations

The present study contributed to understanding the SARS-OV-2 lineage composition of different waves of COVID-19, and their impact on different public health interventions put in place in response to that pandemic in Burundi. Furthermore, the close evolutionary relationships between imported and community-isolated SARS-COV-2 lineages in Burundi imply COVID-19 transmission between Burundi and other countries. Detecting and monitoring the SARS-COV-2 variants informed the design/ adjustment and the implementation of public health countermeasures aimed at limiting the spread of the COVID-19 pandemic in Burundi. We demonstrated that new peaks of COVID-19 cases were mainly associated with newly introduced SARS-COV-2 VOCs. Furthermore, the present study highlighted the implications of travel restrictions relaxation and the virus mutations on the introduction and the subsequent spread of SARS-COV-2 variants in Burundi. Therefore, it is worthy to strengthen the genomic surveillance of SARS-COV-2, enhance the protection by increasing the SARS-COV-2 vaccine coverage, and adjust the public health and social measures ahead of the emergence or introduction of new SARS-COV-2 VOCs in the country.

## Methods

### Study design and population

We conducted a cross-sectional descriptive study on SARS-COV-2 RT-PCR confirmed samples collected in Burundi from May 2021 to January 2022. The study population comprised COVID-19 cases aged ≥ 1 year who tested positive for the SARS-COV-2 at the screening or control test performed at different testing facilities across the country. The study took place at the National Reference Laboratory located in the National Institute of Public Health, Burundi.

### Sampling design

We used a consecutive sampling approach to enroll the study participants, followed by oropharyngeal swab sample collection. After Real-time RT-PCR testing, we selected COVID-19-positive samples with cycle threshold values ≤ 30 for SARS-COV-2 genomic sequencing. Further inclusion criteria included the quantity of the remaining sample after the RT-PCR testing (≥ 1 mL), the availability of metadata, and the storage of the sample at an appropriate temperature (≤ 8 °C for ≤ 48 h or at -80 °C for longer storage). In total, 563 samples had sufficient data and met the criteria for inclusion in the present study.

### Clinical and demographic data collection instruments

We collected the demographic and clinical data using a standardized data collection form designed for COVID-19. The data captured included the age of the patient, gender, province, and commune of residence, date of symptom onset, history of travel in the 14 days preceding the symptom onset, the country visited, date of sample collection, and whether the case is hospitalized or not.

### Sample collection and transport

The oropharyngeal samples were collected from COVID-19 patients at different testing sites across the country, following the Standard Operation Procedures instructions provided by the National Reference Laboratory. The specimens were then labeled, placed in tightly sealable tubes containing the Universal Transport Medium for storage and transported in cooler boxes containing ice packs.

### PCR testing of the samples

The automatic extraction and purification of the SARS-COV-2 RNA were performed on Abbott m2000sp System using the Abbott mSample Preparation System kit (Abbott GmbH & Co. KG, Max-Planck-Ring 2, 65,205 Wiesbaden, Germany). The extracted viral RNA was subsequently subjected to real-time reverse-transcription polymerase chain reaction for cDNA synthesis and amplification of target regions using Abbott Real-Time SARS-CoV-2 amplification reagent kit (Abbott Molecular Inc 1300; East Touhy Avenue Des Plaines, IL 60018 USA).

### Library preparation and whole genome sequencing

The viral genomes used in this study were sequenced by using MinIon at Uganda Virus Research Institute (UVRI), and Illumina at Institut Pasteur de Dakar (IPD), Senegal. The choice of the sequencing platform was based on the preference of the sequencing laboratory and the availability of the sequencing reagents and consumables. Briefly, the nucleic acid was re-extracted and purified using the QIAamp viral RNA extraction kit following the manufacturer’s instructions. The extracted viral RNA was subsequently reverse-transcribed using the Superscript III kit followed by PCR amplification of the resulting cDNA using Entebbe primers, as described elsewhere [30]. After a clean-up step using AMPure XP beads, Agencourt, barcodes, and adapters were successively added followed by amplicons pooling. The DNA library was prepared using the MinIon sequencing kits SQK-LSK109. Normalized DNA libraries were loaded onto MinIon Flow Cell (R9.4.1) and sequenced on a MinIon device [30]. On the other hand, another set of SARS-COV-2 samples was sequenced using the platform Illumina (NextSeq550 (Illumina, San Diego) and Iseq100).

### Bioinformatics analysis

Genome assembly was performed against the reference SARS-COV-2 genome (SARS-CoV-2 isolate Wuhan-Hu-1, complete genome, GenBank accession number NC_045512.2) using the EPI2ME software for sequence reads generated by MinIon whereas the platform EDGE COVID-19 [31]was used for FASTA consensus sequences generation from the sequence reads (FASTQ file) generated through Illumina. The reads generated using Illumina were mapped against the reference genome using the Burrows-Wheeler Aligner (BWA mem) [32]. A threshold >  × 20 was a requirement for a nitrogenic base to be incorporated into the consensus genome. Otherwise, the base was masked as N. The generated FASTA sequences were fed into the PANGOLIN tool [33] and Nextclade [34] to assign the SARS-COV-2 PANGO lineages and clades. Furthermore, from the consensus sequences generated in this study, multiple sequence alignment was performed using MAFFT v7.453 [35, 36] against the reference SARS-COV-2 genome. The sequence alignment was visualized in BioEdit v7.2.5 [37] and subsequently used to infer a maximum likelihood (ML) phylogenetic tree using IQTREE v 2.0.3 [38, 39] and Model Finder to determine the best-fit nucleotide substitution model [40]. We used an Ultrafast Bootstrap of 1000 replicates to assess the ML tree branch supports [41]. The generated ML phylogenetic tree (in a Newick file format) was then fed into Figtree v1.4.2 software for genome visualization. Moreover, the amino acid mutation profiles of SARS-COV-2 genomes identified in Burundi were investigated using the Nextclade tool [42].

### Statistical data analysis

The statistical data from this study were parsed and analyzed in R v4.1.3 [43]. The proportions of different lineages were calculated in R, analyzed against different months of the year 2021 and visualized through bar charts using the ggplot2 package.

## Supplementary Information

**Additional file 1. **

**Additional file 2. **

## Data Availability

The genome sequences generated in this study have been deposited into GISAID EpiCov (Accession numbers: EPI_ISL_2928004-12, EPI_ISL_4949158-212, EPI_ISL_13298627-13298720); https://doi.org/10.55876/gis8.230125db.
