# Supplementary material for: Genomic surveillance of severe acute respiratory syndrome coronavirus 2 in Burundi, from May 2021 to January 2022
Source: BMC Genomics. 2023 Jun 10;24:312. doi: 10.1186/s12864-023-09420-3 (PMC10257533; doi:10.1186/s12864-023-09420-3)
Supplement: Supplementary file 1 — Additional file 1. [file 12864_2023_9420_MOESM1_ESM.docx]

**The sequencing platforms and output details**

| **Sequence ID** | **Sequencing platform** |  | **PANGO Lineage** | **Number of Mutations** | **Coverage (%)** | **Gap** |
| --- | --- | --- | --- | --- | --- | --- |
| 002_07_10_S1 | Illumina |  | AY.122 | 40 | 99.8 | 14 |
| 005_07_10_S4 | Illumina |  | AY.46 | 43 | 98.9 | 13 |
| 006_07_10_S5 | Illumina |  | AY.46 | 42 | 98.7 | 13 |
| 008_08_10_S6 | Illumina |  | AY.46 | 41 | 98.9 | 16 |
| 009_08_10_S7 | Illumina |  | AY.46 | 42 | 99.9 | 13 |
| 010_08_10_S8 | Illumina |  | AY.46 | 42 | 99 | 13 |
| 012_08_10_S10 | Illumina |  | AY.122 | 42 | 98.9 | 13 |
| 013_08_10_S11 | Illumina |  | AY.46 | 39 | 93.2 | 16 |
| 014_08_10_S12 | Illumina |  | AY.46 | 39 | 99 | 13 |
| 015_08_10_S13 | Illumina |  | AY.46 | 37 | 98.9 | 13 |
| 018_08_10_S16 | Illumina |  | AY.46 | 40 | 98.8 | 17 |
| 021_08_10_S20 | Illumina |  | AY.46 | 35 | 97.2 | 22 |
| 022_11_10_S21 | Illumina |  | AY.46 | 42 | 98.8 | 13 |
| 023_11_10_S22 | Illumina |  | AY.46 | 42 | 99.3 | 13 |
| 024_11_10_S23 | Illumina |  | AY.122 | 42 | 99.1 | 13 |
| 026_11_10_S25 | Illumina |  | AY.122 | 43 | 99 | 13 |
| 027_11_10_S26 | Illumina |  | AY.46 | 45 | 99 | 13 |
| 030_11_10_S28 | Illumina |  | AY.46 | 42 | 99 | 16 |
| 101_7_KBR_S80 | Illumina |  | AY.46 | 41 | 99.7 | 22 |
| 114_7_KBR_S82 | Illumina |  | AY.46 | 40 | 99.7 | 22 |
| 116_7_KBR_S78 | Illumina |  | AY.46 | 39 | 99.8 | 22 |
| 128_7_KBR_S90 | Illumina |  | AY.46 | 40 | 99.8 | 22 |
| 129_7_KBR_S68 | Illumina |  | AY.46 | 40 | 99.7 | 22 |
| 130_7_KBR_S79 | Illumina |  | AY.46 | 40 | 99.7 | 22 |
| 137_7_KBR_S73 | Illumina |  | AY.46 | 40 | 99.7 | 22 |
| 138_7_KBR_S100 | Illumina |  | AY.46 | 43 | 99.8 | 22 |
| 140_7_KBR_S89 | Illumina |  | AY.46 | 33 | 98.9 | 16 |
| 141_7_KBR_S74 | Illumina |  | AY.46 | 37 | 99.7 | 22 |
| 144_7_KBR_S76 | Illumina |  | AY.46 | 38 | 99.7 | 22 |
| 145_7_KBR_S84 | Illumina |  | AY.46 | 38 | 99.7 | 22 |
| 148_7_KBR_S88 | Illumina |  | AY.46 | 37 | 99.7 | 22 |
| 150_7_KBR_S65 | Illumina |  | AY.46 | 40 | 99.7 | 22 |
| 151_7_KBR_S94 | Illumina |  | AY.46 | 39 | 99.3 | 22 |
| 162_7_KBR_S92 | Illumina |  | AY.46 | 41 | 98 | 22 |
| 178_7_KBR_S69 | Illumina |  | AY.46 | 39 | 99.8 | 49 |
| 180_7_KBR_S72 | Illumina |  | AY.46 | 40 | 99.1 | 22 |
| BA10_MB_N_S56 | Illumina |  | AY.46 | 40 | 99.7 | 13 |
| BA13_ND_R_S57 | Illumina |  | AY.46 | 35 | 99.8 | 13 |
| BA14_ND_F_S60 | Illumina |  | AY.46 | 39 | 99.7 | 13 |
| BA15_NI_R_S55 | Illumina |  | AY.46 | 41 | 98.5 | 13 |
| BA161_IR_I_S61 | Illumina |  | AY.46 | 40 | 99.8 | 13 |
| BA26_NYA_J_S50 | Illumina |  | AY.122 | 39 | 99.8 | 25 |
| BA28_NA_J_S49 | Illumina |  | AY.122 | 42 | 99.5 | 19 |
| BUN_416_S94 | Illumina |  | BA.1 | 41 | 96.1 | 36 |
| BUN_417_S90 | Illumina |  | BA.1 | 40 | 87.2 | 36 |
| BUN_422_S25 | Illumina |  | B.1.1.529 | 25 | 82.9 | 27 |
| BUN_424_S82 | Illumina |  | BA.1 | 41 | 95.7 | 39 |
| BUN_425_S85 | Illumina |  | BA.1 | 44 | 95.4 | 36 |
| BUN_433_S96 | Illumina |  | BA.1.1 | 31 | 86.2 | 39 |
| BUN_434_S74 | Illumina |  | BA.1 | 41 | 92.1 | 36 |
| BUN_436_S97 | Illumina |  | BA.1.1 | 30 | 84.8 | 36 |
| BUN_437_S91 | Illumina |  | BA.1 | 43 | 88.9 | 36 |
| BUN_438_S45 | Illumina |  | BA.1.1 | 27 | 84 | 21 |
| BUN_439_S1 | Illumina |  | B.1.1.529 | 20 | 80.5 | 20 |
| BUN_443_S69 | Illumina |  | B.1.1.529 | 24 | 80.6 | 20 |
| BUN_445_S77 | Illumina |  | BA.1 | 40 | 87 | 21 |
| BUN_448_S30 | Illumina |  | BA.1 | 29 | 85.5 | 36 |
| BUN_449_S68 | Illumina |  | BA.1 | 20 | 82.7 | 27 |
| BUN_450_S42 | Illumina |  | BA.1 | 40 | 87.9 | 27 |
| BUN_451_S11 | Illumina |  | BA.1 | 29 | 86.1 | 36 |
| BUN_452_S27 | Illumina |  | BA.1 | 38 | 88.6 | 21 |
| BUN_453_S62 | Illumina |  | BA.1 | 42 | 93.7 | 36 |
| BUN_454_S66 | Illumina |  | BA.1 | 33 | 84.5 | 36 |
| BUN_456_S51 | Illumina |  | B.1.1.529 | 24 | 83 | 22 |
| BUN_457_S5 | Illumina |  | BA.1 | 39 | 86.4 | 27 |
| BUN_458_S14 | Illumina |  | BA.1 | 26 | 82.1 | 27 |
| BUN_461_S67 | Illumina |  | BA.1 | 33 | 81.4 | 36 |
| BUN_462_S36 | Illumina |  | BA.1 | 25 | 81.4 | 21 |
| BUN_463_S52 | Illumina |  | C.37.1 | 19 | 84.1 | 21 |
| BUN_464_S46 | Illumina |  | BA.1 | 28 | 82.5 | 17 |
| BUN_465_S12 | Illumina |  | BA.1 | 42 | 95.3 | 39 |
| BUN_466_S70 | Illumina |  | BA.1 | 48 | 97.1 | 39 |
| BUN_472_S43 | Illumina |  | BA.1.1 | 37 | 84.7 | 36 |
| BUN_475_S59 | Illumina |  | BA.1 | 27 | 83.5 | 20 |
| BUN_476_S16 | Illumina |  | B.1.1.529 | 36 | 81.9 | 27 |
| BUN_477_S15 | Illumina |  | BA.1 | 37 | 82.7 | 36 |
| BUN_480_S57 | Illumina |  | B.1.619 | 24 | 81.2 | 27 |
| BUN_483_S34 | Illumina |  | BA.1 | 41 | 82.7 | 39 |
| BUN_485_S44 | Illumina |  | B.1 | 27 | 83.3 | 21 |
| BUN_488_S65 | Illumina |  | BA.1 | 43 | 88.8 | 36 |
| BUN_489_S84 | Illumina |  | BA.1 | 41 | 84.2 | 27 |
| BUN_490_S22 | Illumina |  | BA.1 | 34 | 80.9 | 21 |
| BUN_491_S71 | Illumina |  | BA.1.1 | 32 | 81.2 | 36 |
| BUN_492_S3 | Illumina |  | BA.1 | 40 | 92.9 | 36 |
| BUN_495_S18 | Illumina |  | BA.1 | 40 | 89.6 | 36 |
| BUN_496_S98 | Illumina |  | BA.1.1 | 26 | 82.3 | 36 |
| BUN_499_S56 | Illumina |  | BA.1 | 44 | 94.6 | 36 |
| BUN_500_S86 | Illumina |  | BA.1 | 41 | 85.3 | 36 |
| BUN_501_S81 | Illumina |  | B.1.1.529 | 33 | 81.7 | 20 |
| BUN_503_S6 | Illumina |  | B.1.1.529 | 27 | 82.1 | 27 |
| BUN_505_S35 | Illumina |  | BA.1.1 | 33 | 80.5 | 36 |
| BUN_506_S75 | Illumina |  | B.1 | 26 | 80 | 30 |
| BUN_507_S61 | Illumina |  | B.1 | 23 | 84.9 | 22 |
| BUN_508_S60 | Illumina |  | BA.1 | 40 | 86.4 | 36 |
| BUN_509_S64 | Illumina |  | BA.1 | 40 | 85.9 | 36 |
| BUN_511_S88 | Illumina |  | BA.1.1 | 37 | 81.9 | 30 |
| BUN_512_S28 | Illumina |  | BA.1.1 | 37 | 80.7 | 36 |
| BUN_513_S95 | Illumina |  | B.1.1 | 23 | 81 | 10 |
| BUN_519_S33 | Illumina |  | BA.1 | 40 | 92.8 | 36 |
| G10_N2_JB_S41 | Illumina |  | AY.46 | 36 | 97.5 | 13 |
| G12_IT_TE_S35 | Illumina |  | AY.122 | 43 | 98.5 | 13 |
| G14_IT_OL_S43 | Illumina |  | AY.46 | 40 | 98.8 | 13 |
| G15_MA_G0_S39 | Illumina |  | AY.122 | 36 | 98.2 | 15 |
| G16_BI_AL_S37 | Illumina |  | AY.46 | 33 | 95.5 | 16 |
| G19_AK_ST_S45 | Illumina |  | AY.122 | 44 | 98 | 13 |
| MA16_IR_Cl_S42 | Illumina |  | AY.122 | 40 | 98.3 | 13 |
| MA17_NI_JC_S31 | Illumina |  | AY.122 | 40 | 98.1 | 13 |
| Burundi\|EPI_ISL_2928004 | MinIon |  | B.1.351 | 26 | 99.4 | 18 |
| Burundi\|EPI_ISL_2928005 | MinIon |  | B.1.351 | 30 | 99.7 | 18 |
| Burundi\|EPI_ISL_2928006 | MinIon |  | B.1.617.2 | 34 | 99.6 | 12 |
| Burundi\|EPI_ISL_2928007 | MinIon |  | B.1.1.7 | 38 | 99.5 | 18 |
| Burundi\|EPI_ISL_2928008 | MinIon |  | B.617.2 | 34 | 99.8 | 12 |
| Burundi\|EPI_ISL_2928009 | MinIon |  | B.1.351 | 28 | 99.6 | 18 |
| Burundi\|EPI_ISL_2928010 | MinIon |  | B.1.351 | 28 | 99.6 | 18 |
| Burundi\|EPI_ISL_2928011 | MinIon |  | B.1.617.2 | 36 | 99.4 | 12 |
| Burundi\|EPI_ISL_2928012 | MinIon |  | B.1.351 | 27 | 98.8 | 18 |
| Burundi\|EPI_ISL_4949158 | MinIon |  | B.1.617.2 | 32 | 99.3 | 12 |
| Burundi\|EPI_ISL_4949159 | MinIon |  | AY.46 | 37 | 99.3 | 12 |
| Burundi\|EPI_ISL_4949160 | MinIon |  | B.1.617.2 | 29 | 98.8 | 12 |
| Burundi\|EPI_ISL_4949161 | MinIon |  | AY.46 | 34 | 99.2 | 12 |
| Burundi\|EPI_ISL_4949162 | MinIon |  | B.1.617.2 | 24 | 98.8 | 12 |
| Burundi\|EPI_ISL_4949164 | MinIon |  | B.1.617.2 | 30 | 98.8 | 12 |
| Burundi\|EPI_ISL_4949165 | MinIon |  | B.1.617.2 | 33 | 99 | 12 |
| Burundi\|EPI_ISL_4949166 | MinIon |  | AY.46 | 33 | 98.8 | 12 |
| Burundi\|EPI_ISL_4949167 | MinIon |  | AY.46 | 33 | 99.4 | 12 |
| Burundi\|EPI_ISL_4949168 | MinIon |  | AY.46 | 33 | 98.8 | 12 |
| Burundi\|EPI_ISL_4949169 | MinIon |  | AY.46 | 32 | 98.7 | 12 |
| Burundi\|EPI_ISL_4949170 | MinIon |  | AY.46 | 31 | 98.7 | 12 |
| Burundi\|EPI_ISL_4949171 | MinIon |  | AY.46 | 33 | 98.8 | 12 |
| Burundi\|EPI_ISL_4949172 | MinIon |  | B.1.617.2 | 29 | 99.3 | 12 |
| Burundi\|EPI_ISL_4949173 | MinIon |  | B.1.617.2 | 32 | 98.8 | 12 |
| Burundi\|EPI_ISL_4949174 | MinIon |  | B.1.617.2 | 33 | 99 | 12 |
| Burundi\|EPI_ISL_4949175 | MinIon |  | B.1.617.2 | 36 | 99.6 | 12 |
| Burundi\|EPI_ISL_4949176 | MinIon |  | B.1.617.2 | 36 | 99.6 | 12 |
| Burundi\|EPI_ISL_4949177 | MinIon |  | B.1.617.2 | 30 | 99.4 | 12 |
| Burundi\|EPI_ISL_4949178 | MinIon |  | B.1.617.2 | 29 | 99.6 | 12 |
| Burundi\|EPI_ISL_4949179 | MinIon |  | B.1.617.2 | 34 | 99.4 | 12 |
| Burundi\|EPI_ISL_4949180 | MinIon |  | B.1.617.2 | 38 | 99.4 | 12 |
| Burundi\|EPI_ISL_4949181 | MinIon |  | B.1.617.2 | 36 | 99.4 | 12 |
| Burundi\|EPI_ISL_4949182 | MinIon |  | B.1.617.2 | 36 | 99.5 | 12 |
| Burundi\|EPI_ISL_4949183 | MinIon |  | B.1.617.2 | 36 | 99.5 | 12 |
| Burundi\|EPI_ISL_4949184 | MinIon |  | B.1.617.2 | 39 | 99.5 | 12 |
| Burundi\|EPI_ISL_4949185 | MinIon |  | B.1.617.2 | 34 | 99.4 | 12 |
| Burundi\|EPI_ISL_4949186 | MinIon |  | B.1.617.2 | 36 | 99.5 | 12 |
| Burundi\|EPI_ISL_4949187 | MinIon |  | B.1.617.2 | 32 | 99.5 | 12 |
| Burundi\|EPI_ISL_4949188 | MinIon |  | B.1.617.2 | 37 | 99.4 | 12 |
| Burundi\|EPI_ISL_4949189 | MinIon |  | B.1.617.2 | 33 | 99.5 | 12 |
| Burundi\|EPI_ISL_4949190 | MinIon |  | B.1.617.2 | 33 | 99.5 | 12 |
| Burundi\|EPI_ISL_4949191 | MinIon |  | B.1.617.2 | 33 | 99.4 | 12 |
| Burundi\|EPI_ISL_4949192 | MinIon |  | B.1.617.2 | 33 | 99.6 | 12 |
| Burundi\|EPI_ISL_4949193 | MinIon |  | B.1.617.2 | 33 | 99.4 | 12 |
| Burundi\|EPI_ISL_4949194 | MinIon |  | B.1.617.2 | 33 | 99.6 | 12 |
| Burundi\|EPI_ISL_4949195 | MinIon |  | B.1.617.2 | 33 | 99.5 | 12 |
| Burundi\|EPI_ISL_4949196 | MinIon |  | B.1.617.2 | 33 | 99.4 | 12 |
| Burundi\|EPI_ISL_4949197 | MinIon |  | B.1.617.2 | 33 | 99.4 | 12 |
| Burundi\|EPI_ISL_4949198 | MinIon |  | B.1.617.2 | 32 | 99.4 | 12 |
| Burundi\|EPI_ISL_4949199 | MinIon |  | B.1.617.2 | 30 | 99.4 | 12 |
| Burundi\|EPI_ISL_4949200 | MinIon |  | B.1.617.2 | 33 | 99.5 | 12 |
| Burundi\|EPI_ISL_4949201 | MinIon |  | B.1.617.2 | 32 | 99.6 | 12 |
| Burundi\|EPI_ISL_4949202 | MinIon |  | B.1.617.2 | 33 | 99.4 | 12 |
| Burundi\|EPI_ISL_4949203 | MinIon |  | B.1.617.2 | 33 | 99.5 | 12 |
| Burundi\|EPI_ISL_4949204 | MinIon |  | AY.46 | 34 | 99.4 | 12 |
| Burundi\|EPI_ISL_4949205 | MinIon |  | AY.46 | 37 | 99.4 | 12 |
| Burundi\|EPI_ISL_4949206 | MinIon |  | AY.46 | 35 | 99.7 | 12 |
| Burundi\|EPI_ISL_4949207 | MinIon |  | AY.46 | 35 | 99.4 | 12 |
| Burundi\|EPI_ISL_4949208 | MinIon |  | AY.46 | 35 | 99.4 | 12 |
| Burundi\|EPI_ISL_4949209 | MinIon |  | AY.46 | 34 | 99.6 | 12 |
| Burundi\|EPI_ISL_4949210 | MinIon |  | AY.46 | 35 | 99.4 | 12 |
| Burundi\|EPI_ISL_4949211 | MinIon |  | B.1.617.2 | 32 | 99.4 | 12 |
| Burundi\|EPI_ISL_4949212 | MinIon |  | B.1.617.2 | 36 | 99.4 | 12 |
