# Supplementary material for: Genomic surveillance of severe acute respiratory syndrome coronavirus 2 in Burundi, from May 2021 to January 2022
Source: BMC Genomics. 2023 Jun 10;24:312. doi: 10.1186/s12864-023-09420-3 (PMC10257533; doi:10.1186/s12864-023-09420-3)
Supplement: Supplementary file 2 — Additional file 2. [file 12864_2023_9420_MOESM2_ESM.docx]

**The predominant amino acid changes carried by different SARS-COV-2 lineages identified in Burundi from May 2021 to January 2022.**

|  | **AMINO ACID CHANGES PER LINEAGE (GISAID CLADES)** | | | | | | | |
| --- | --- | --- | --- | --- | --- | --- | --- | --- |
| **Lineage (Clade)** | **AY.43 (G)** | **AY.46 (GK)** | **B.1.1.529 (GRA)** | **BA.1 (GRA)** | **BA.1.1 (GRA)** | **B.1.617.2 (GK)** | **B.1.1.7 (GRY)** | **B.1.351 (GH)** |
|  | A381T | D377Y | E31del | P132H | K38R | D377Y | D3L | P13S |
| N protein | R203M | D63G | P13L | E31del | G204R | D63G | G204R | T205I |
|  |  | G215C | R32del | P13L | P13L | R203M | R203K |  |
|  |  | R203M | S33del | S33del | R203K |  | A570D |  |
|  |  |  |  |  | R32del |  |  |  |
|  |  |  |  |  | S33del |  |  |  |
| Spike protein | D614G | D614G | A67V | A67V | A67V | A222V | D1118H | A701V |
|  | L981F | D950N | D614G | D614G | D614G | D614G | D614G | D215G |
|  | N679K | E156G | D796Y | D796Y | D796Y | D950N | H69del | D215G |
|  | N969K | F157del | L981F | G142D | G142D | E156G | N501Y | D614G |
|  | P681H | G142D | N764K | H655Y | H655Y | F157del | P681H | D80A |
|  | Q954H | L452R | N856K | H69del | H69del | G142D | S982A | E484K |
|  |  | P681R | N969K | L981F | L981F | L452R | T716I | K417N |
|  |  | R158del | Q954H | N679K | N679K | P681R | V70del | L18F |
|  |  | T19R | T547K | N764K | N764K | R158del | Y144del | L244del |
|  |  | T478K | T95I | N856K | N856K | S698L |  | L54F |
|  |  |  |  | N969K | N969K | T19R |  | N501Y |
|  |  |  |  | P681H | P681H | G142D |  | S1252F |
|  |  |  |  | Q954H | Q954H | T478K |  | A243del |
|  |  |  |  | T547K | T547K |  |  | L244del |
|  |  |  |  | T95I | T95I |  |  | L54F |
|  |  |  |  | V143del | V143del |  |  |  |
|  |  |  |  | V70del | Y144del |  |  |  |
|  |  |  |  | Y144del | Y145del |  |  |  |
|  |  |  |  | Y145del |  |  |  |  |
| NSP2 |  |  | F356V |  |  | G671S | L550F | T85I |
|  |  |  |  |  |  |  | T412I |  |
| NSP3 | L1266I | A488S | L1266I |  | A1892T | P822L | A890D | K837N |
|  | S1265del | P1228L | S1265del |  | L1266I |  | I1412T |  |
|  |  | P1469S | V1150A |  | S1265del |  | I397V |  |
|  |  |  |  |  |  |  | N1104S |  |
| NSP4 | T492I | T492I | T492I |  | S312N | A446V | T183I |  |
|  |  | V167L |  |  | T492I |  | T64I |  |
| NSP5 |  |  | P132H |  | P132H | D153G |  | K90R |
| NSP6 | I189V | T77A | G107del | I189V | G107del | T181I | F108del | F108del |
|  | T77A |  | L105del | L105del | L105del | V149A | G107del | G107del |
|  |  |  | S106del | S106del | S106del |  | S106del | S106del |
| NSP12 | P323L | G671S | P323L |  |  | G671S | P323L | P323L |
|  |  | P323L |  |  |  | P323L | V720A |  |
|  |  |  |  |  |  | T141A |  |  |
| NSP13 |  | P77L |  |  |  | P77L |  |  |
| NSP14 | A394V | A394V |  |  | I42V |  |  |  |
|  | I42V |  |  |  |  |  |  |  |
